# Individual variability in the nuclei of the human superior olivary complex

**DOI:** 10.1007/s00429-025-03005-4

**Published:** 2025-08-25

**Authors:** Yahya Farid, Bryan Lukyanenko, Sandra F. Witelson, Joan S. Baizer

**Affiliations:** 1 Department of Physiology and Biophysics, Jacobs School of Medicine and Biomedical Sciences, University at Buffalo, 123 Sherman Hall, South Campus, Buffalo, NY 14214 USA; 2 Department of Psychiatry and Behavioural Neurosciences, Michael G. DeGroote School of Medicine, McMaster University, Hamilton, ON L8S 4K1 Canada

**Keywords:** Auditory system, Sound localization, Cochlear implants, Binaural hearing, Immunohistochemistry, Medial superior olive, Lateral superior olive

## Abstract

The superior olivary complex (SOC) receives auditory information from the cochlear nuclei. In nonhuman mammals, the SOC contains three nuclei: the lateral and medial superior olives (LSO, MSO) and the medial nucleus of the trapezoid body (MNTB). There are also periolivary neurons that are assigned to different nuclei in different mammals. The configuration of the SOC in the human differs from that in other species. The LSO is less well-defined; some authors do, and others do not, find an MNTB, and different authors recognize different periolivary nuclei. We have studied the organization of the human SOC using Nissl and immunostained sections of 12 brains from the Witelson Normal Brain Collection. We found an MSO in all cases although it varied in rostro-caudal extent. We did not consistently see a grouping of neurons consistent with an LSO in Nissl sections. Calbindin (CB) is expressed in neurons of the MNTB in several species. We found CB-immunoreactive (ir) cells in all human cases, some in the expected location of the MNTB, however these CB-ir neurons varied in number and location among cases. The variability in SOC configuration suggests there may also be individual variability in sound localization, a major function mediated by the SOC.

## Introduction

**Fig. 1:**
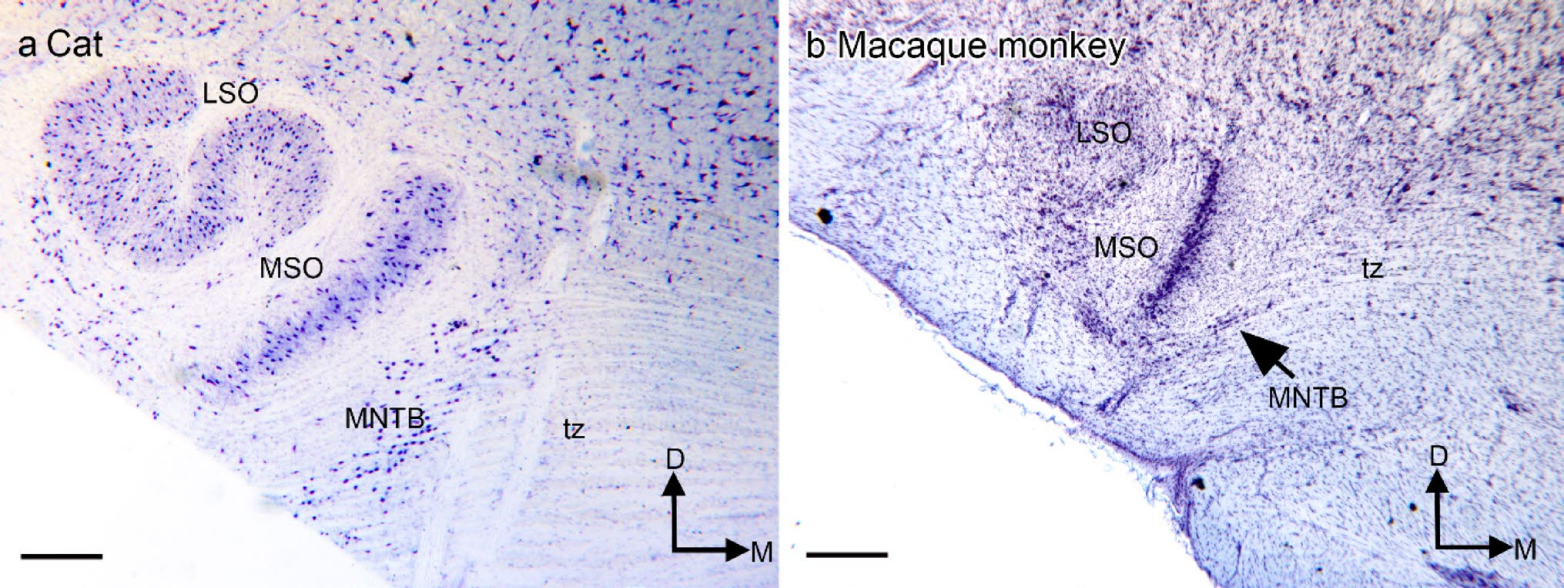
**a** The SOC in the cat shown on a Nissl-stained section from a celloidin-embedded brain. The LSO, MSO and MNTB are clearly defined structures. **b** The SOC on a celloidin-embedded Nissl-stained section from a macaque monkey. The MSO is a cell column similar to that in the cat, the boundaries of the LSO are less marked and there are many fewer cells (MNTB) among the fibers of the tz. Scale bars a, b = 500 μm. Abbreviations in Table 1, for all figures

The auditory nerve conveys information from the cochlea to the dorsal (DCN) and ventral (VCN) cochlear nuclei (CN; Cohen et al. 1972; Powell and Cowan 1962; Fekete et al. 1984; all abbreviations in Table 1). These nuclei in turn project to a number of targets including the nuclei of the superior olivary complex, the inferior colliculus and the cerebellum (Baydyuk et al. 2016; Cant and Casseday 1986; Shneiderman and Henkel 1985; SOC; Glendenning et al. 1985; Glendenning et al. 1991; review of auditory pathways in Häusler and Levine 2000; Illing et al. 2000; Kil et al. 1995; Huang et al. 1982; Stotler 1953; Warr 1969). The SOC is critical for sound localization, with neurons that respond to differences in the signals received by the two ears (Goldberg and Brown 1969; Tollin 2003; Grothe et al. 2010; Yin 2002; Keating and King 2013; Illing et al. 2000; reviews in Moore 1991). There have been many studies of the SOC in different mammalian species, including the cat and several rodents (a few examples among many: Alvarado et al. 2004; Ollo and Schwartz 1979; Grothe and Park 2000; Irvine 1986; Bazwinsky et al. 2005; Kil et al. 1995; Kiss and Majorossy 1983; Schwartz 1977; Schofield and Cant 1991; Rincón et al. 2024; Rietzel and Friauf 1998; Saldaña et al. 2009). These studies agree that the SOC includes three well-defined nuclei, the medial (MSO) and lateral (LSO) superior olivary nuclei, and the medial nucleus of the trapezoid body (MNTB). In addition, there are neurons, called periolivary neurons, distributed around these structures. Some studies describe periolivary neurons as organized into nuclei but the number, sizes and locations of proposed nuclei vary among species (Ollo and Schwartz 1979; mouse; Sommer et al. 1993; rat; Saldaña et al. 2009; rat; Kelley et al. 1992; chinchilla; Grothe and Park 2000; bat; Kuwabara and Zook 1999; gerbil; Schofield and Cant 1991; guinea pig; Spirou and Berrebi 1997; cat; Matsubara 1990; cat; Spirou and Berrebi 1996; cat; Thompson and Schofield 2000; cat; Irvine 1986; Strominger et al. 1977; chimpanzee; Bazwinsky et al. 2003; humans). Figure 1 shows the SOC in Nissl sections of two different species, the cat (Fig. 1a) and the macaque monkey (Fig. 1b). In the cat, the MSO, LSO and the MNTB are very well demarcated in a Nissl stain. In the monkey, the MSO is well-defined, the LSO is present but has a different configuration than in the cat, and there are many fewer darkly-stained neurons in the trapezoid body (tz) compared to the cat (compare Fig. 1a and b).

**Table 1:** Abbreviations

6n	Fibers of the abducens nerve
7 N	Nucleus of the seventh cranial nerve (facial)
CB	Calbindin
ChAT	Choline acetyltransferase
CR	Calretinin
CV	Cresyl violet
GLYR	Glycine receptor
GLYT2	Neuronal glycine transporter
-ir	immunoreactive
LSO	Lateral superior olive
MNTB	Medial nucleus of the trapezoid body
Ms	Mouse
MSO	Medial superior olive
NPNFP	Nonphosphorylated neurofilament protein
PMI	Post-mortem interval
PV	Parvalbumin
Rb	Rabbit
SOC	Superior olivary complex
SPON	Superior paraolivary nucleus
tz	trapezoid body

The relative sizes and organization of the SOC components are different in humans than in other species (reviews in Moore 2000; Glendenning and Masterton 1998; Irvine 1986; Moore and Moore 1971). The MSO has been identified in all studies of the human SOC; it is consistently described as a column of neurons, 1–3 neurons wide (Strominger and Hurwitz 1976; Kulesza 2007, 2014; Moore 2000; Bazwinsky et al. 2003; Hilbig et al. 2009). However, results for the LSO, the MNTB and periolivary cell groups are less consistent. Some studies find a distinct LSO whereas others describe cell clusters in the expected region of the LSO but not a discrete nucleus (Strominger and Hurwitz 1976; Moore and Moore 1971; Hilbig et al. 2009; Irving and Harrison 1967; Kulesza 2007; Bazwinsky et al. 2003; Irvine 1986). In humans, the existence of an MNTB has been controversial. Several studies reported that the MNTB is absent in humans (Moore and Moore 1971; Strominger and Hurwitz 1976; Weinrich et al. 2018; Bazwinsky et al. 2003; Hilbig et al. 2009; Moore 2000); others argue that it does exist (review in Kulesza and Grothe 2015; Richter et al. 1983; Weinrich et al. 2018; Kulesza 2008, 2014). In humans, there is no periolivary nucleus that is consistently identified (Bazwinsky et al. 2003; Kulesza 2008, 2014; Moore 2000).

What might account for these discrepancies among studies? First, there may have been differences among studies in tissue quality. A key factor in tissue preservation in human studies is the time between death and immersion of the brain in fixative, the postmortem interval, PMI, and PMI varies among studies. Different studies also use different procedures for fixing and storing first the brains and then the cut sections. Finally, different laboratories may use different staining protocols. All of these parameters can affect the staining quality and hence the appearance of the slides that are available for analysis. Second, different authors may have used different criteria for identifying candidate MNTB neurons. Yet another idea is that the different results may reflect individual variability in the size and shape of the nuclei of the SOC. For example, it is possible that the MNTB may exist in some brains but not in others; the LSO may be a clearly defined nucleus in some brains but only cell clusters in others. We have shown individual variability in the sizes, shapes and rostro-caudal extent of other human brainstem nuclei, for example the arcuate nucleus of the medulla and the principal nucleus of the inferior olive (Baizer et al. 2011b, 2018b, 2021).

We have studied the organization of the SOC in humans from the perspective of individual variability. We used Nissl-stained sections to define major nuclei. We also analyzed sections immunostained for several different markers to identify cell and axonal features of the SOC. Neurons in the ventral cochlear nucleus and their axons express CR (Baizer et al. 2018a; Kulesza 2014); sections immunostained for CR can visualize CN inputs to the SOC (for example Fig. 6 in Kulesza 2014). Since neurons of the MNTB receive input from the cochlear nuclei (review in Cant and Benson 2003), CR-ir input should help in localizing them. We found that nonphosphorylated neurofilament protein (NPNFP) is a useful marker for somata and dendrites of neurons in the MSO. Principal neurons of the MNTB in multiple species express the calcium-binding protein calbindin D-28 K (CB), (cat, Matsubara 1990; Adams and Mugnaini 1990; bats, Zettel et al. 1991; chinchilla, Kelley et al. 1992; guinea pig, Caicedo et al. 1996); we used CB-ir to search for candidate MNTB neurons. Finally, neurons of the MNTB in other species provide glycinergic input to the MSO, LSO, and periolivary neurons (Rampon et al. 1996; Spirou and Berrebi 1997; Smith et al. 1998; Bledsoe et al. 1990; Spangler et al. 1985; Adams and Mugnaini 1990; Kuwabara and Zook 1992; Grothe and Sanes 1993). We looked for evidence of glycinergic neurotransmission in the human SOC using antibodies to glycine receptors and the glycine transporter.

## Materials and methods

### Human brainstems

We studied twelve human brainstems from the Witelson Normal Brain Collection; the details of subject selection and tissue acquisition were described in Witelson and McCulloch (1991). Subjects were patients diagnosed with metastatic cancer; at the time of enrollment into the study up to the time of death they had no diagnosed neurological disease. They then underwent neuropsychological testing focused on standard and experimental dichotic listening tasks. Each subject underwent standard audiological testing, had hearing levels within normal limits for each ear and did not wear hearing aids. Patients were then followed medically and periodically screened for the development of neurologic dysfunction. At death, brains were removed by pathologists, fixed in formalin, and examined for neuropathology. We selected cases with short postmortem intervals (PMIs) and no documented neurological dysfunction in life or neuropathology noted after death.

Table 2 shows the case number, age, sex, and PMI (in hours) of the human brain specimens we studied. We have previously reported data on protein expression in various brainstem nuclei of several of these cases, including the CN, the vestibular nuclei, the inferior olive, and the arcuate nucleus (Baizer et al. 2007, 2011a, c, 2013, 2014a, b, 2018a, b, 2021, 2024; Baizer and Broussard 2010).

**Table 2:** Cases from the Witelson normal brain collection

Case	Age	Sex	PMI (hr)
125	57	m	5
155	50	f	9
158	51	m	1
164	45	f	3
166	65	f	3
167	55	f	2
168	69	m	3
169	70	m	2
176	71	f	3
178	53	m	2
180	54	m	2
183	69	m	2

### Histological procedures

Our histological methods have been described previously (Baizer et al. 2007, 2013; Baizer and Broussard 2010). Briefly, tissue blocks containing the brainstem and cerebellum were dissected away from the fixed cerebrum, and all tissue was stored in 10% formalin. We further separated the brainstem and cerebellum and then cryoprotected the brainstems, first in 15%, then in 30% sucrose in 10% formalin. Prior to sectioning, we made a small slit along one side of the ventral brainstem to allow identification of left and right sides of the brain. Forty µm-thick frozen sections of the brainstem were cut on an American Optical (AO) sliding microtome in a plane transverse to the brainstem. All sections were collected and stored in 5% formalin in large plastic compartment boxes, with 5 consecutive sections in each compartment. The boxes were stored at 4°C. For each case, we first stained sets of sections no more than 2 mm apart with a Nissl stain, Cresyl Violet (CV), following a standard protocol (LaBossiere and Glickstein 1976). For subsequent studies of different brainstem structures (Baizer et al. 2007, 2011a, c, 2013, 2018a, b, 2021) additional sections were CV-stained as needed to define the sizes of structures of interest. We also used the CV-stained sections to identify sections for immunohistochemistry (IHC) to describe the neurochemical characteristics of neurons in the different brainstem nuclei. We used the same staining protocols for sections from each case.

For this project, we initially analyzed archival CV and immunostained sections through the SOC. We stained additional sections with CV to determine the rostro-caudal extent of the MSO (in mm, ± 200 μm) in each case. Kulesza (2008; Fig. 1) showed the MNTB ventromedial to the MSO along almost its entire rostrocaudal extent. We therefore immunostained sections at the level of the MSO with CB to identify candidate MNTB neurons. Table 3 shows the total number of sections analyzed to determine the rostrocaudal extent of the MSO for each case. The “misc. IHC” column includes sections that had been immunostained for NPNFP, the glycine transporter, glycine receptors, glutamate receptors, PV, nNOS or ChAT in the course of published or pilot studies. The total number of sections includes all sections on which the MSO was found as well as the sections just rostral and just caudal to the MSO.

**Table 3:** MSO sections analyzed

Case	CV	CB	CR	misc. IHC	total	MSO extent (mm)
125	11	1	0	0	12	3.9
155	14	6	5	6	31	4.8
158	11	5	5	4	25	5.7
164	11	8	6	9	34	4.6
166	8	9	3	2	22	4.0
167	7	2	0	0	9	4.1
168	16	3	1	5	25	3.7
169	10	4	11	10	35	5.6
176	8	3	2	2	15	4.6
178	14	2	0	0	16	5.9
180	15	4	2	4	25	6.0
183	12	1	0	0	13	4.9

### Antibodies and IHC

All IHC was performed on free-floating sections. Sections were rinsed in phosphate buffered saline (PBS, all rinses were 3 × 10 min). Sections were then treated with an antigen retrieval (AR) protocol. Each section was placed in a separate small glass jar with 20 ml of pH = 6 citrate buffer. The jars were heated in a water bath at 85° C for 30 min. The jars were removed from the bath and cooled to room temperature. Sections were removed from the jars, rinsed in PBS and nonspecific label blocked by incubating sections in a solution of phosphate buffered saline (PBS), 1% Triton-X 100, 1% bovine serum albumin and 1.5% normal serum (from the appropriate Vector Elite kit). The primary antibody was added to the blocking solution, and sections incubated overnight at 4 °C on a tissue rocker. Further processing was with the Vector “ABC” method using the appropriate Vector Elite kit (mouse or rabbit; Vector Laboratories, Burlingame, CA), followed by visualization with a 3,3’-diaminobenzidine (Sigma-Aldrich now Thermo Fisher) protocol, giving brown staining, or a glucose-oxidase modification of the protocol giving gray-black staining (Shu et al. 1988; Van der Gucht et al. 2006). Sections were mounted on gelled slides, dehydrated in 70%, 95% and 100% alcohol, cleared in Xylene and coverslipped with Permount (Fisher Scientific). Table 4 shows the primary antibodies and dilutions used.

**Table 4:** Antibodies and dilutions

Antigen	Source, Catalogue #	Host	Type	Dilution
CB	Proteintech, 14479-1-AP	Rb	polyclonal	1:1000
CR	Chemicon, AB 5054	Rb	polyclonal	1:1000-1:3000
GLYR	Abcam, ab23809	Rb	polyclonal	1:1000
GLYT2	Santa Cruz, sc-390,090	Ms	monoclonal	1:200
NPNFP	Covance, SMI32	Ms	monoclonal	1:1000

### Data analysis and photography

We examined sections with a Leitz Dialux 20 light microscope and captured digital images (1600 × 1200 pixels) with a SPOT Insight Color Mosaic camera. Brightness, contrast and color of the images were adjusted, and figures assembled, with Adobe Photoshop software (San Jose, CA).

## Results

### Configuration and components of the SOC

We found that the MSO was the only SOC cell group that was well-defined in Nissl sections in all cases. Its rostrocaudal extent varied among cases (3.7–6.0 mm, x̄ =4.8, Table 3). While there were many neurons around the MSO, they did not consistently appear in well-defined clusters. In every case, CR-ir defined a large area surrounding the cell column of the MSO, presumably the area receiving input from the CN. We found neurons that were immunostained for CB, but the numbers, appearance and locations of these CB-ir neurons varied among cases. There was evidence of glycinergic input to the SOC. We will illustrate these major findings using data from eight cases.

**Fig. 2:**
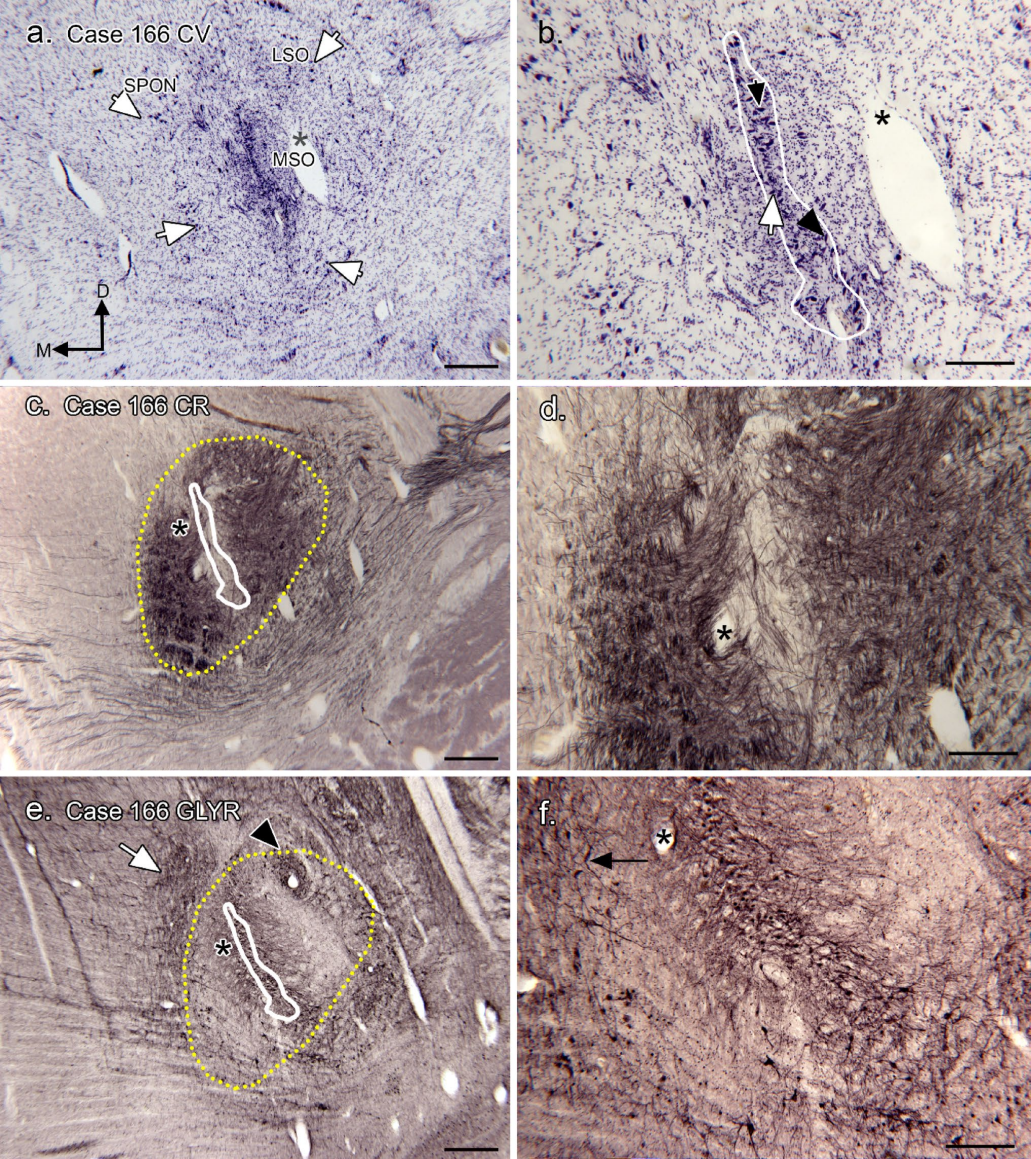
The SOC in Case 166. **a** Nissl-stained section from the middle of the rostro-caudal extent of the MSO. The asterisk is an alignment point for the higher magnification image in b. The arrows show examples of neurons surrounding the MSO neuronal column. The label “LSO” shows the region in which the LSO is typically identified, and the label “SPON” the region of the superior paraolivary nucleus (Kulesza 2008). The directional arrows indicate medial (M) and dorsal (D) with the same orientation for all panels. **b** The MSO neuron column is outlined in white. The white arrow indicates a bipolar/fusiform neuron with its long axis perpendicular to the long axis of the MSO neuron column. The black arrow shows a stellate/multipolar cell, and the black arrowhead a fusiform cell whose long axis is oblique to the axis of the MSO neuronal column. The asterisk is an alignment point for the image in a. **c** CR-ir on a section about 200 μm caudal to the one in a. The central region of the MSO, where the somata are found, has very little label (white outline). The dense CR-ir region is outlined in yellow. The asterisk is an alignment point for the higher magnification image in d. **d** There are a few CR-ir fibers crossing the region of the MSO neuron column. **e** Section adjacent to the one shown in b immunostained for the glycine receptor (GLYR). The column of MSO cell bodies is outlined in white; and the CR-ir region by the yellow dotted line. The arrowhead indicated a small region of dense GLYR-ir dorsolateral to the MSO, and the arrowhead a patch of label medial and dorsal to the MSO cell column. This asterisk is an alignment point for the image in f. (In this case, there is also immunostaining of an unknown protein in cell nuclei resulting in the many small black dots visible on the higher magnification image). **f** GLYR-ir on the MSO dendrites and several medial neurons (arrow). Scale bars: a, b, c, d = 500 μm; e, f, g = 100 μm

Figures 2a, b show a Nissl-stained section (Case 166). The MSO is a very well-defined structure; it appears as a narrow column of neurons (outlined in white in Fig. 2b; this outline is then superimposed on the immunostained sections (Fig. 2c, e; the same convention is followed in the other figures). The width of the MSO neuronal column varies from 1 to about 3 neurons. There are two major soma shapes: bipolar neurons (Fig. 2b, white arrow, black arrowhead) and multipolar neurons (Fig. 2b, black arrow). Many bipolar neurons are oriented with their long axes roughly perpendicular to the axis of the column of somata (Fig. 2b, arrow); others are seen at more oblique angles (Fig. 2b, arrowhead).

There are neurons surrounding the MSO cell column (Fig. 2a, examples at white arrows). We show the expected locations of the superior paraolivary nucleus (SPON) and the LSO based on the report of Kulesza (2014); on the section shown here the borders of these nuclei are not well-defined. There are scattered neurons in the fibers of the tz but these are not limited to the expected location of the MNTB (and compare with Fig. 1a, b.). Figure 2c, d show CR-ir on an adjacent section. CR-ir defines an oval-shaped region of dense label (outlined in yellow) with a very lightly labeled region dividing it in half. The CR-ir region lateral to the MSO is larger than the region medial to it. Superimposing the MSO outline (from Fig. 2b) onto the CR-ir section shows that this lightly stained region corresponds to the MSO cell column, suggesting a lack of CR-ir input onto the MSO somata. The higher magnification image of the CR-ir region (Fig. 2d) shows that there are a few CR-ir fibers crossing the region of the MSO cell column. We examined the CR-ir fibers in the trapezoid body (tz) and in the expected location of the MNTB and were not able to identify endings characteristic of the calyces of Held (Kulesza 2014) in this, or the other cases. The regions in which the LSO and SPON have been found were within the CR-ir area. Figure 2e, f shows immunoreactivity to the glycine receptor (GLYR) on a section adjacent to the one in a. There is GLYR-ir label overlapping the MSO neuronal cell column, with label of the dendrites. There is a patch of label (Fig. 2e, black arrowhead) dorsolateral to the MSO; this patch of label may define an LSO. There is also a small patch of immunolabel dorsomedial to the MSO (white arrowhead) in a region that may overlap the region identified as the SPON. Figure 2f shows immunolabel of a periolivary neuron (black arrow) medial to the MSO cell column.

**Fig. 3:**
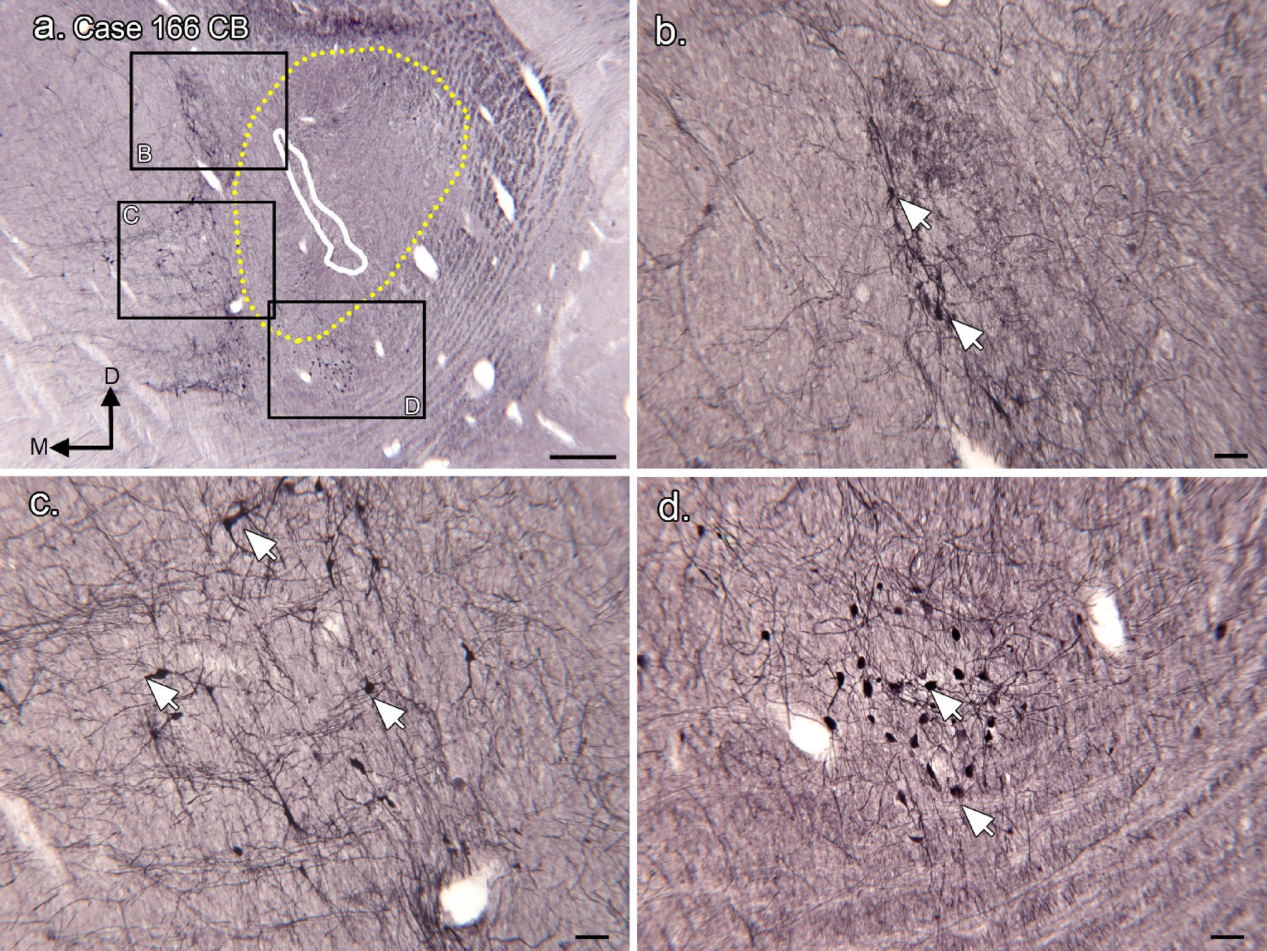
Calbindin immunoreactive cells in the SOC of Case 166. **a** The MSO cell column is outlined in white (based on Fig. 1a, b), and the CR-ir input region in yellow (based on Fig. 1c). The labeled rectangles show the locations of the higher magnification images in b, c, d. The directional arrows indicate medial (M) and dorsal (D) with the same orientation for all panels. **b** CB-ir neurons and dendrites dorsomedial to the MSO. **c** Scattered CB-ir neurons lateral to the MSO. **d** Small cluster of CB-ir neurons (arrows) ventral to the MSO. Scale bars: a = 500 μm, b, c, d = 100 μm

We next asked if immunoreactivity to CB provided evidence for a distinct MNTB. Figure 3a shows CB-ir on a section about 200 μm caudal to the one in Fig. 2a. The white outline shows the MSO cell column (from Fig. 1b); the dotted yellow outline shows the region of CR-ir input, (from Fig. 2c). There are CB-ir cell clusters dorsomedial (Fig. 3b), ventromedial (Fig. 3c) and ventral (Fig. 3d) to the MSO. The appearance of the CB-ir neurons varies with location. In Fig. 3b there is extensive staining of dendrites of scattered cells; in Fig. 3c the CB-ir neurons are more widely spaced with a variety of soma shapes (arrows). Figure 3d shows a cluster of closely spaced small neurons with round or oval somata and very little staining of dendrites. In this case, CB-ir does not define a single neuronal population in the expected location of the MNTB.

**Fig. 4:**
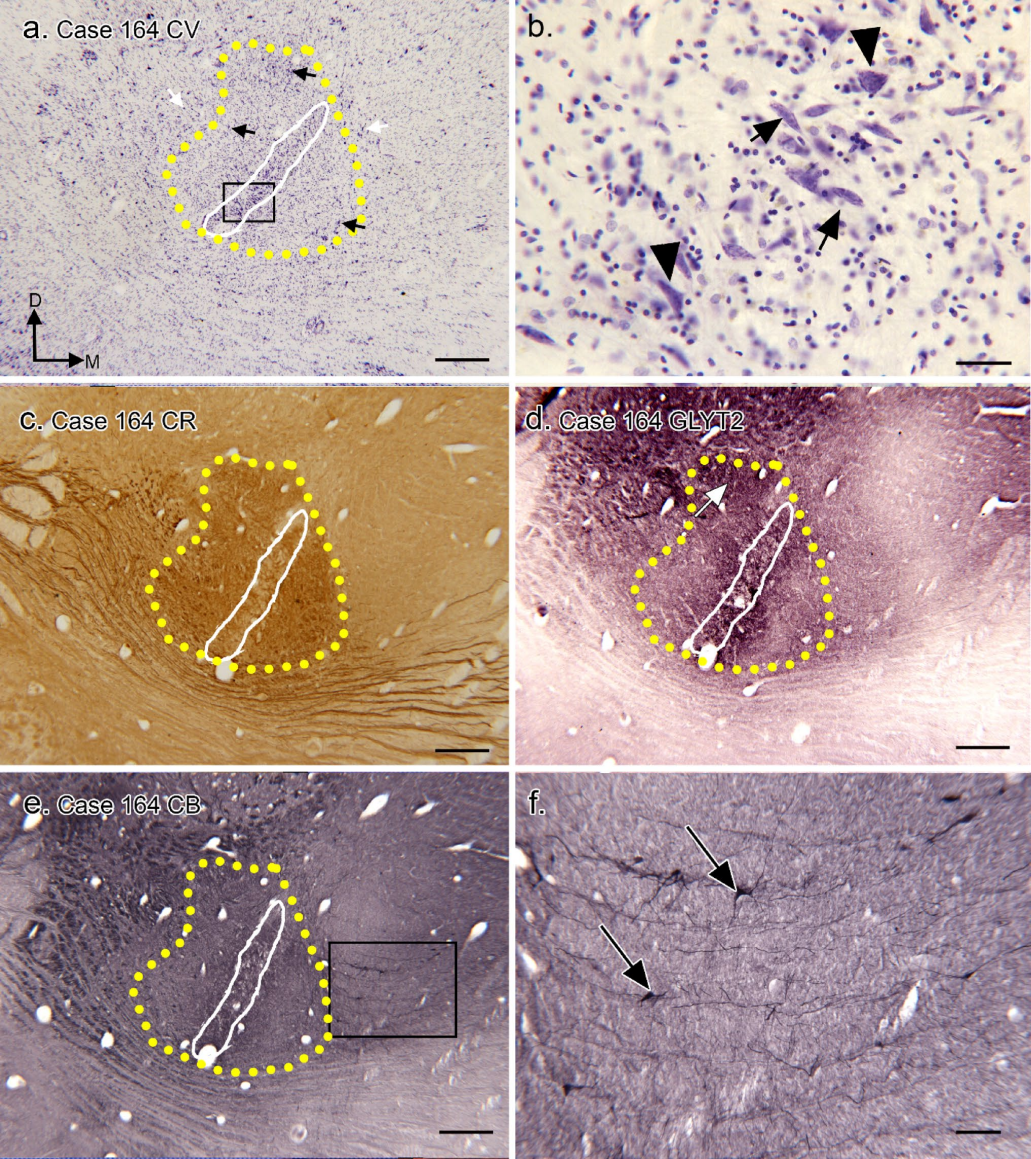
The SOC in Case 164. **a** Nissl-stained section including the MSO; the MSO column is outlined in white. The section is about 1600 μm rostral to the caudal start of the MSO. The yellow dotted line shows the region of dense CR-ir input, (determined from the image in c). The rectangle shows the location of the higher magnification image of MSO somata in b. The black arrows show cells outside of the MSO cell column; the most dorsal arrow shows a cell cluster in the expected location of the LSO. The directional arrows indicate medial (M) and dorsal (D) with the same orientation for all panels. **b** The somata of the MSO are bipolar (examples at arrows) and multipolar (examples at arrowheads). **c** CR-ir on a section about 200 μm caudal to the section in a. There is a region of dense CR-ir density, outlined by the dotted yellow line; on either side of the MSO somata (white outline). **d** Immunostaining for the glycine transporter on a section adjacent to the section in c shows GLYT2-ir overlapping the MSO somata (white outline) with lighter immunostaining in the CR-ir region (yellow dotted line). The white arrow indicates darker staining in the expected location of the LSO. **e** CB-ir on a section adjacent to the section in d. The rectangle shows the location of the higher magnification image in f. **f** CB-ir somata medial to the MSO (examples at arrows). Scale bars: a, b, c, d, e = 500 μm, f = 100 μm

The data from Case 164 (Fig. 4) support the basic observations in Case 166. The MSO cell column is clearly defined (Fig. 4a, white outline, the section illustrated is 400 μm caudal to the middle of the MSO). The MSO column of neurons is composed of bipolar (Fig. 4b, examples at black arrows) and multipolar (Fig. 4b, black arrowheads) neurons. There are neurons around the MSO cell column (Fig. 4a, examples at 3 black arrows). The most dorsal black arrow shows a cell cluster that may correspond to the LSO; the other two arrows show neurons that are not in clearly-defined clusters. There is an asymmetrical region of CR-ir surrounding the MSO cell column (Fig. 4c, yellow outline). GLYT2-ir suggests glycinergic input to the MSO cell column and dendrites (Fig. 4d) as well as to a region dorsolateral to the MSO. Immunostaining for CB (Fig. 4e, f) shows only a few CB-ir neurons (Fig. 4f, arrows), and these are medial to the MSO.

**Fig. 5:**
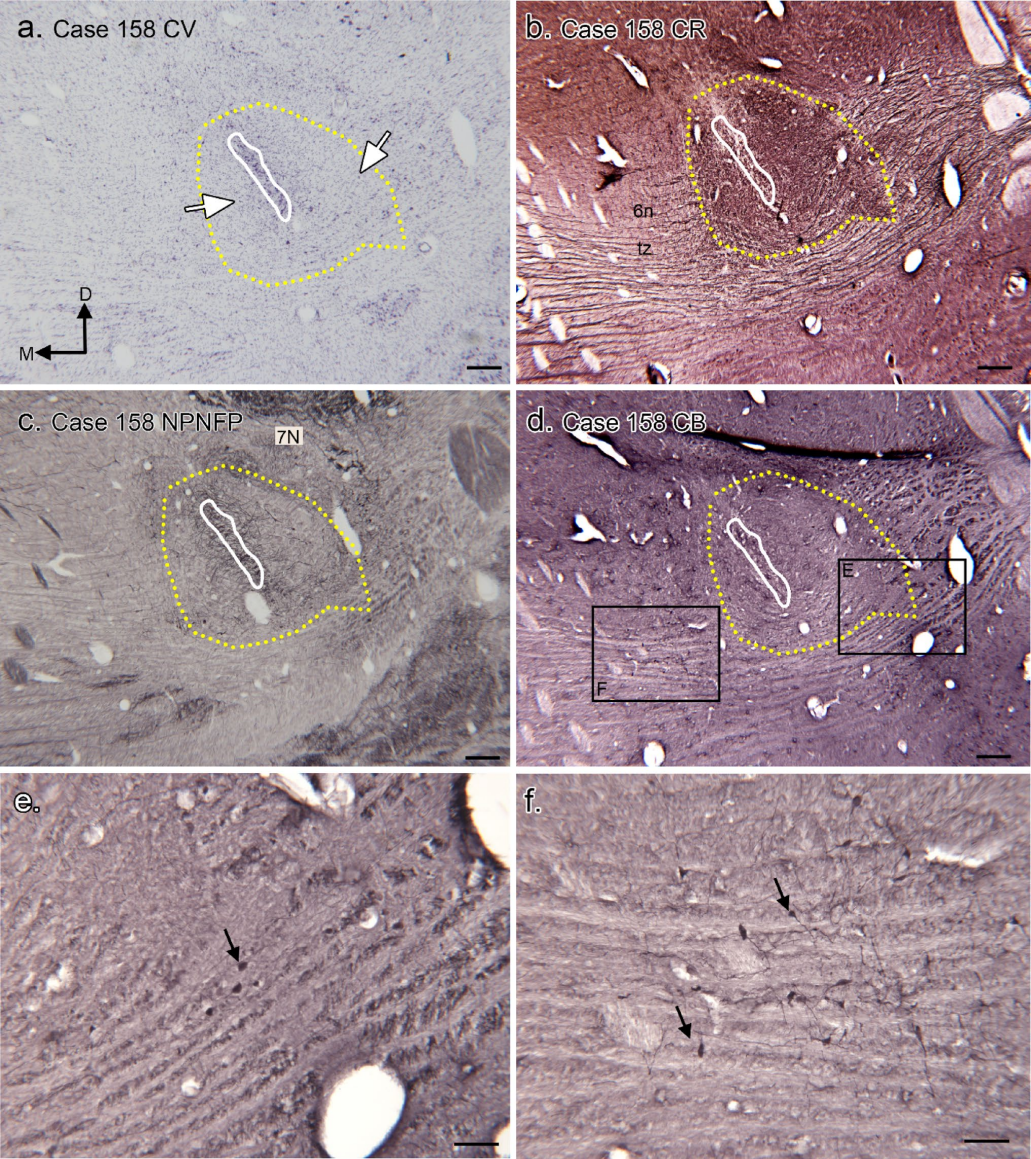
The SOC in Case 158. **a** Nissl-stained section through the MSO at the middle of its rostro-caudal extent. The seventh nerve nucleus (7 N) is dorso-lateral to it. The MSO cell column is outlined in white, the CR-ir area in yellow. The two white arrows indicate examples of neurons around the MSO cell column. The directional arrows indicate medial (M) and dorsal (D) with the same orientation for all panels. **b** CR-ir on a section adjacent to the one in a. The dense CR-ir around the MSO is outlined in yellow and the region of MSO somata (from a) in white. **c** NPNFP-ir on a section about 0.6 mm rostral to the one in a; the MSO somata and CR-ir dense regions are outlined in white and yellow respectively. **d** CB-ir section adjacent to the section in a. The labeled rectangles show the locations of the images in e and f. **e** Small CB-ir somata lateral to the MSO, example at arrow. **f** CB-ir somata medial to the MSO. Scale bars a, b, c, d = 500 μm; e, f = 100 μm

Figure 5 illustrates the SOC in Case 158. Figure 5a shows a Nissl section from the middle of the rostro-caudal extent of the MSO. The MSO neuron column is outlined in white. As in the other cases, there are neurons distributed around the MSO (examples at white arrows) but there are no clearly-defined cell clusters dorsolateral (LSO) or ventromedial (MNTB) to the MSO, nor are there any other distinct periolivary nuclei. As in the other cases, CR-ir defines a region around the MSO cell column (Fig. 5b, yellow outline) that extends farther laterally than medially. NPNFP-ir labels the MSO dendrites (Fig. 5c), and there is heavy immunolabel in 7 N. Figure 5d shows a section immunostained for CB; there are scattered CB-ir neurons lateral (Fig. 5e) and ventromedial (Fig. 5f) to the MSO; the medial neurons are well-outside the CR-ir region.

**Fig. 6:**
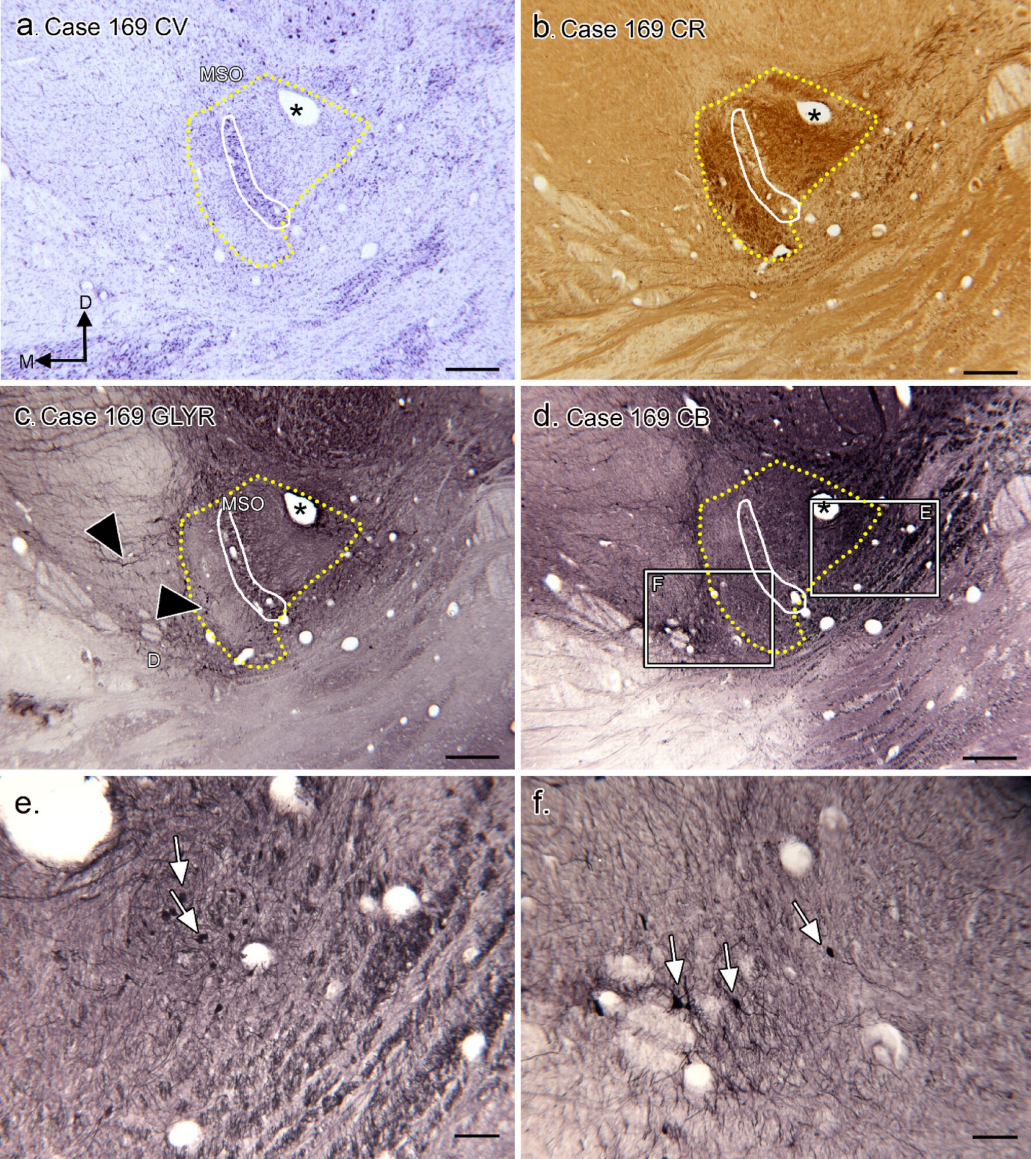
The SOC in Case 169. **a**. Nissl-stained section about 400 μm rostral to the caudal limit of the MSO. The MSO cell column is outlined in white, the CR-ir region in yellow. The asterisk is an alignment point for the images in b - d. The directional arrows indicate medial (M) and dorsal (D) with the same orientation for all panels. **b** CR-ir section adjacent to the section in a. The MSO neuron column (white) is lightly stained, with dark immunostaining medially and laterally (CR-ir region outlined by yellow dotted line). **c** GLYR-ir on a Sect. 400 μm caudal to the section in a. There is immunolabel of the MSO cell column as well as of neurons and processes outside of the CR region (examples at arrowheads). **d** CB-ir on a section adjacent to the section in c. The asterisk is an alignment point for a-c. The labeled rectangles show the locations of the images in e and f. **e** Small cluster of CB-ir neurons (white arrows) located within the dense CR-ir region. **f** Scattered CB-ir neurons (examples at white arrows) located medial to the CR-ir region. Scale bars: a - d = 500 μm; e, f = 100 μm

In Case 169 (Fig. 6) there is a very distinct MSO (Fig. 6a; the section is about 0.8 mm rostral to the caudal start of the MSO), and scattered periolivary neurons that do not group into distinct nuclei. The MSO is surrounded by a region of CR-ir input (Fig. 6b, yellow dotted line) that extends further laterally than medially. Figure 6c shows an adjacent section immunostained with an antibody for the glycine receptor. There is GLYR-ir on the MSO cell column. Outside of the cell column the immunolabel is heavier lateral than medial to the MSO; GLYR-ir is found on elements (arrowheads) both inside and outside the CR-ir region. There are CB-ir neurons both lateral (Fig. 6e, white arrows) and medial (Fig. 6f, white arrows) to the MSO cell column. The appearance of the CB-ir neurons in the two locations is different. One set of neurons has small round somata (Fig. 6e) and the second set consists of larger multipolar cells (Fig. 6f).

**Fig. 7:**
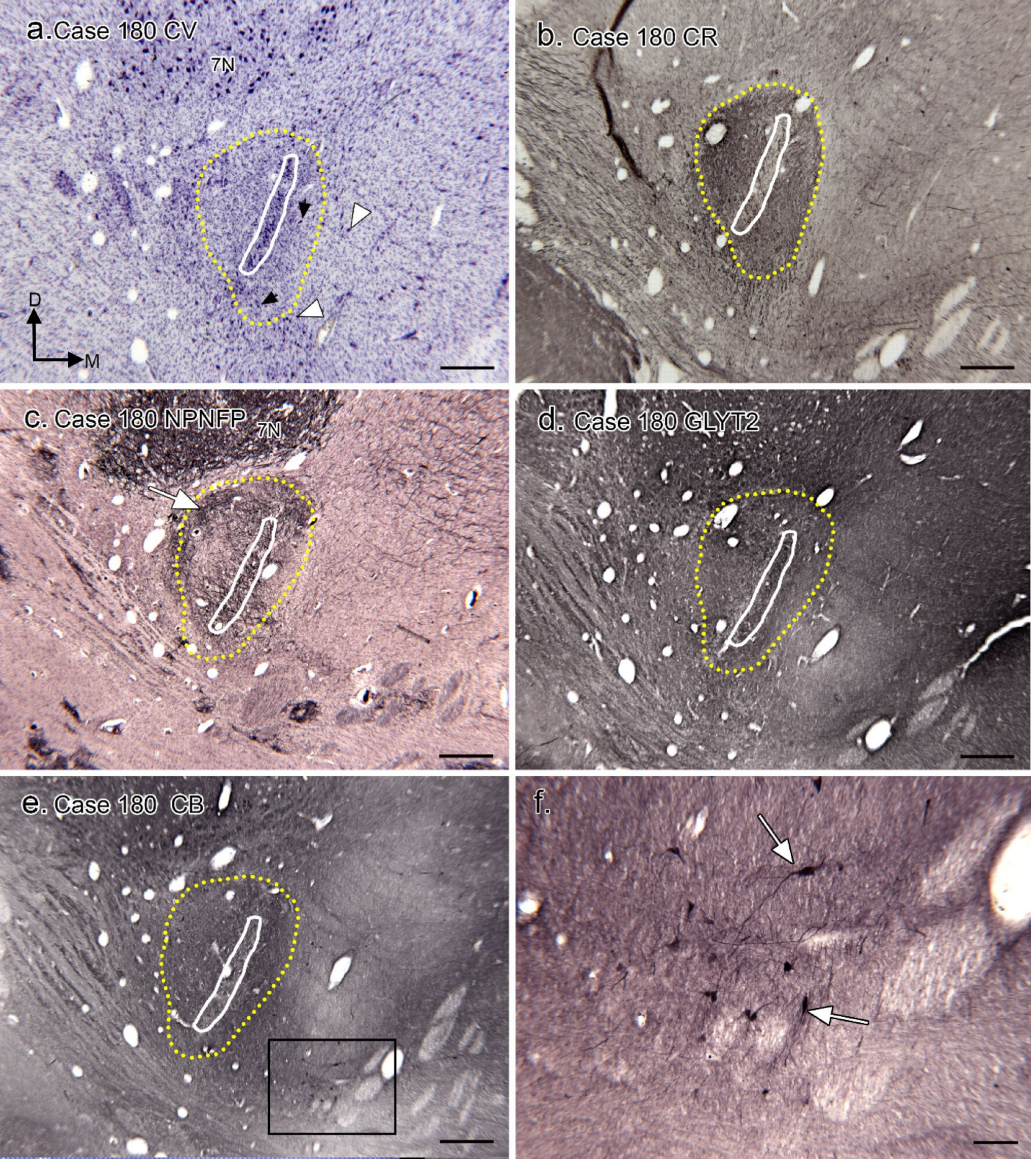
The SOC in Case 180. **a** Nissl-stained Sect. 2 mm caudal to the center of the rostro-caudal extent of the MSO showing the MSO cell column (white outline, arrow; and the region of CR-ir (yellow outline). The seventh nerve nucleus (7 N) is seen dorsolateral to the MSO. The directional arrows indicate medial (M) and dorsal (D) with the same orientation for all panels. **b** CR-ir on a section adjacent to the one in Fig. 6a showing dense label on either side of the MSO cell column (yellow dotted line). **c** NPNFP immunolabel on a section about 200 μm rostral to the one in b. The MSO somata and dendrites are well-labeled; there is very dark immunostaining in 7 N. The MSO cell column is outlined in white, the region of heavy CR-ir in yellow. The white arrow indicates a region of darker immunostaining lateral and dorsal to the MSO. **d** GLYT2 immunolabel on a section about 400 μm rostral to the one in a, with the MSO cell column (outlined in white) immunolabeled. **e** Immunolabel for CB on a section adjacent to the one in d. The cell column is outlined in white, the CR area in yellow. The rectangle shows the location of the image in F which shows scattered CB-ir neurons (examples at arrows) ventromedial to the MSO. Scale bars: a - e = 500 μm; f = 100 μm

**Fig. 8:**
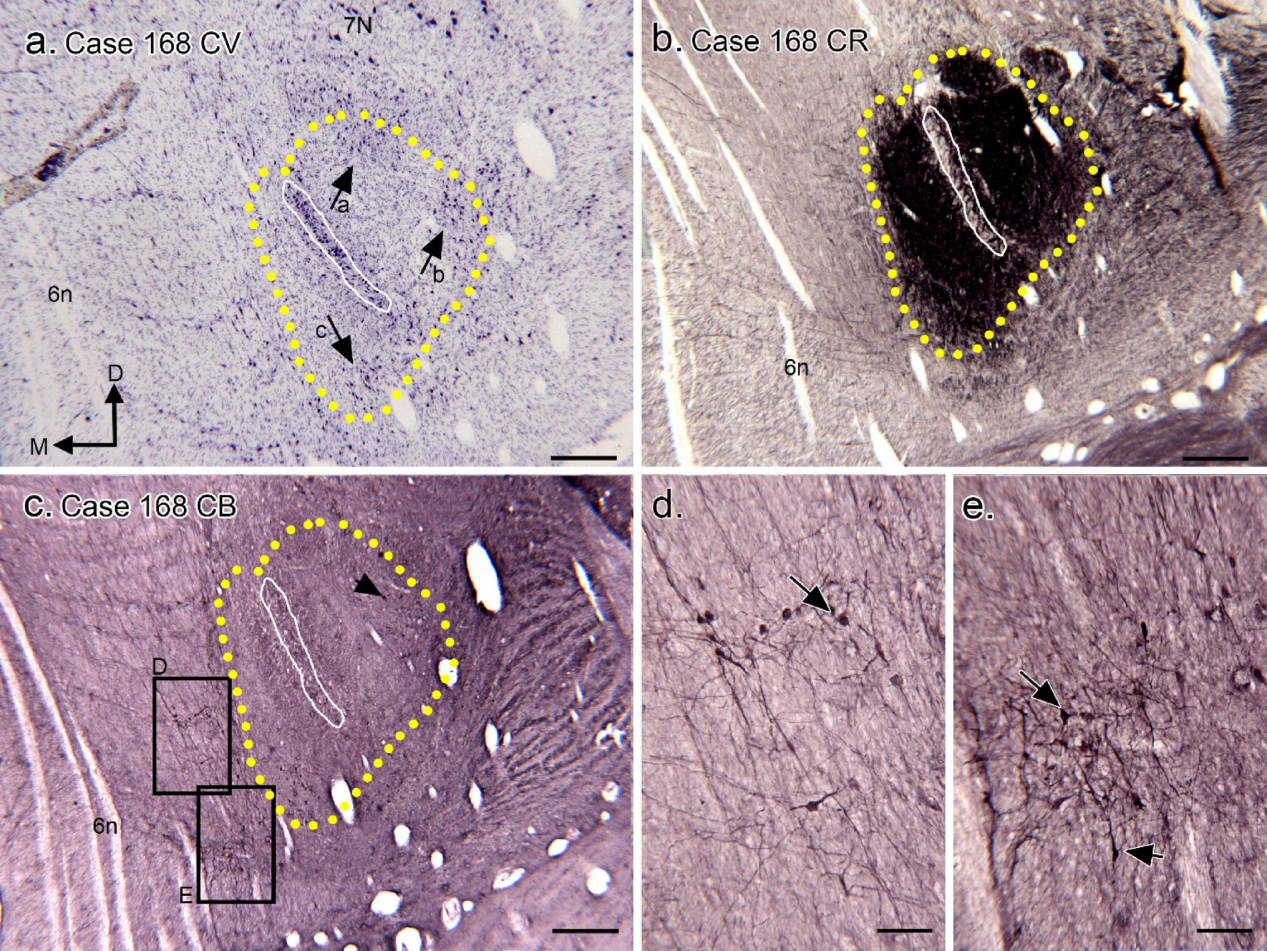
The SOC in Case 168. **a** Nissl-stained section showing the MSO neuron column (outlined in white) and the CR-ir dense surrounding area (yellow outline based on the image in b). The black arrows show cell clusters dorsal, dorsolateral and ventromedial to the cell MSO column. This section is about 800 μm rostral to the caudal limit of the MSO. The directional arrows indicate medial (M) and dorsal (D) with the same orientation for all panels. **b** CR-ir around the MSO (somata column outlined in white) on a section about 400 μm caudal to the one in a. **c** CB-ir on a section about 200 μm rostral to the section in a. The labeled rectangles show the locations of images in d, e. **d** CB-ir neurons (examples at arrows) medial to the MSO. **e** CB-ir neurons ventromedial to the MSO. Note the variety of CB-ir soma shapes and sizes. Scale bars: a- c = 500 μm; d, e = 100 μm

Data from Case 180 support the basic results. Figure 7a shows a Nissl-stained section with the MSO cell column (white outline) and CR-ir area (yellow outline). There are scattered neurons around it both within (black arrows) and outside (white arrowheads) the CR-ir area. This section is about 2 mm caudal to the rostro-caudal center of the MSO. Figure 7b shows the CR-ir area around the MSO, with little label over the MSO cell column and the area lateral to the MSO slightly larger than the area medial to it. Figure 7d shows NPNFP-ir in the MSO dendrites. There is evidence for glycinergic input to the MSO and regions around it (Fig. 7d); this input is denser dorsal to the MSO than ventral to it. In this case there are CB-ir neurons ventro-medial to the MSO (Fig. 7e and f, white arrows). Data from Case 168 (Fig. 8) again show the variability in the numbers and distribution of CB-ir neurons around the MSO. Figure 8a shows the MSO neuron column (white outline) and Fig. 8b the area of CR-ir input (yellow outline) around the cell column. As in the other cases, there are neurons around the MSO cell column, some grouped in clusters (Fig. 8a, black arrows). In this case, there are CB-ir neurons both within (Fig. 8c, example at black arrow), and outside of the CR-ir region (Figs. 8d, e).

**Fig. 9:**
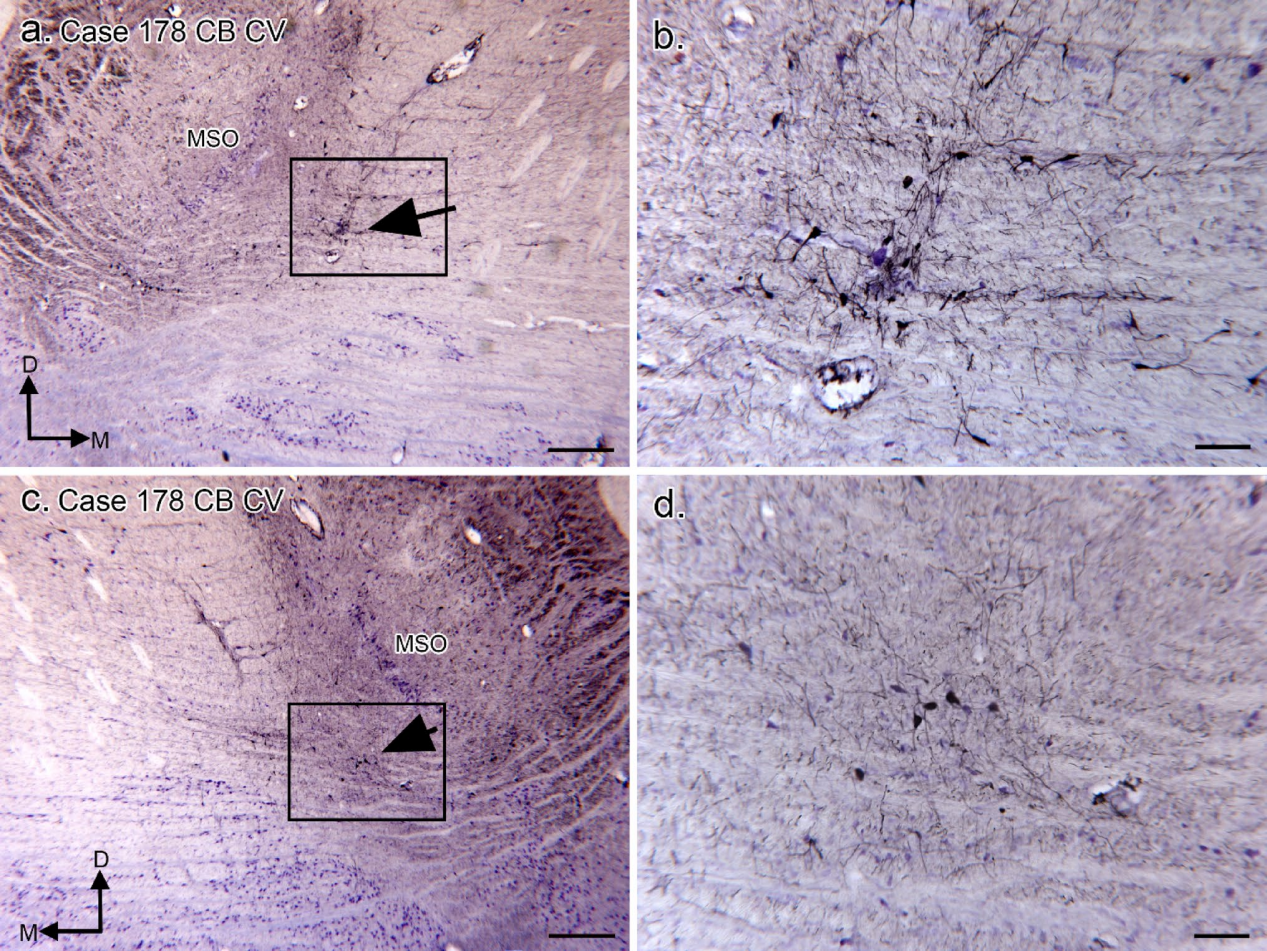
The SOC in Case 178 shown on a section immunostained for CB and lightly counterstained for CV. **a** The MSO neurons are lightly stained. There are CB-ir neurons medial and ventral to the MSO; the arrow indicates one cluster of CB-ir neurons ventromedial to the MSO. The rectangle shows the location of the higher-magnification image in b. **b** Scattered CB-ir neurons and dendrites. **c** The MSO on the right side of the section. The arrow indicates a small cluster of CB-ir neurons, and the rectangle the location of the image in d. d. There are CB-ir neurons and processes ventromedial to the MSO, but not as many as in a. Scale bars a, c = 500 μm; b, d = 100 μm

We have found differences between left and right sides of the brain in the size and shape of other brainstem nuclei, notably the principal nucleus of the inferior olive and the arcuate nucleus of the medulla (Baizer et al. 2011b, 2021). Figure 9 shows the MSO on the left (Fig. 9a, b) and right MSO (Fig. 9c, d) a section that was immunostained for CB and then lightly counterstained with CV. There are CB-ir neurons ventromedial to the MSO (in the predicted location of the MNTB) on both sides (Fig. 9b, d) with more neurons on the left (Fig. 9b) than on the right (Fig. 9d).

## Discussion

We have studied the organization of the superior olivary complex in twelve human cases using Nissl and immunostained sections. There are several major similarities among cases. First, in Nissl-stained sections, the MSO is a very distinct and clearly identified nucleus, although its rostro-caudal extent varies among cases. The dendrites of MSO neurons are immunolabeled with NPNFP. Immunoreactivity for CR shows a region around the MSO with very dense immunostaining, defining the area of CR input from the ventral cochlear nuclei. There are small variations in the size and shape of this area among cases, but it is present in each. Nissl-stained sections show scattered neurons on all sides of the MSO, but there is variability among cases in the arrangements and clusters of these neurons. There are neurons immunoreactive for CB in each case; there are differences among cases in the size, shape, numbers and distribution of these neurons. There is no consistent cluster of CB-ir neurons among the fibers of the tz. Lastly, immunoreactivity for the glycine receptor and the glycine transporter suggests glycinergic input to the MSO and periolivary neurons. We will consider first how these results compare with other studies of humans and then consider the possible functional significance of these individual differences.

### MSO and LSO

Our results on the MSO are in general agreement with prior studies in humans (Bazwinsky et al. 2003; Hilbig et al. 2009; Kulesza 2007, 2008; Moore 2000; Strominger and Hurwitz 1976). We did, however see individual variability in the rostrocaudal extent of the MSO.

An LSO is identified in human studies but descriptions of its size and shape vary. Kulesza (2007) described the LSO as beginning 700 μm rostral to the start of the MSO and continuing for about 3 mm; over part of its extent it was composed of two clusters of cells. (Strominger and Hurwitz 1976) recognized an LSO composed of multiple clusters of cells rather than a single nucleus. Moore (2000) identified a small, oval LSO in humans. We found cell clusters lateral to the MSO in three cases (Cases 164 Fig. 3; Case 166, Figs. 1 and 2 and Case 168 Fig. 7), but no int two other cases (Case 158, Fig. 4; Case 180, Fig. 6). The differences among studies in the size and shape of the LSO are in agreement with our results, suggesting that defining the extent and boundaries of the LSO may be difficult in Nissl stains. Our results suggest individual variability in the organization of this nucleus.

### MNTB

What do our data contribute to the debate about the existence of the MNTB in humans? We found Nissl-stained neurons ventromedial to the MSO among the fibers of the tz in all cases, but the number and distribution varied among cases. We also found CB-ir neurons around the MSO, but again, the numbers and locations of these neurons varied among cases. Many were found outside the expected location of the MNTB, and we did not see a consistent grouping of these cells in among the fibers of the tz where the MNTB is found. These results are consistent with studies in several species that have reported CB-ir neurons in various regions of the SOC, e.g. ventrolateral to the LSO (cat, Matsubara 1990; Fig. 1); in the LNTB (Spirou and Berrebi 1997), in periolivary neurons (bat, Zettel et al. 1991; chinchilla, Kelley et al. 1992). In humans, CB-ir neurons have been found in the LNTB and SPON (Kulesza 2014). Indeed, Hilbig et al. (2009) suggested that the cells that Kulesza (2008) assigned to the MNTB might instead be periolivary cells. It is possible, however, that at least some of the scattered CB-ir neurons serve the same functions as neurons in the MNTB. This idea is compatible with the suggestion of Moore and Moore (1971) who concluded “The trapezoid nucleus, on the other hand, becomes a group of lightly stained and diffusely scattered cells in both apes and man.”

### Periolivary neurons and nuclei

We did not find consistent cell clusters corresponding to defined periolivary nuclei. In humans, different numbers of periolivary nuclei are recognized in different studies, e.g. dorsal, lateral and ventral periolivary nuclei in Bazwinsky et al. (2003); the SPON and LNTB in Kulesza (2014) and lateral, dorsal, rostral, and ventral periolivary nuclei in Moore (2000). These discrepancies may reflect the difficulty of defining these nuclei in Nissl sections. It is possible that distinct nuclei could be better defined by neurochemical markers as suggested by our data for NPNP (Fig. 7c) and GLYR (Fig. 2e). However, simply describing the neurons in the CR-ir region around the MSO as “periolivary neurons” is most consistent with the data.

We found a large area of CR-ir around the MSO, delineating the region of input from the ipsilateral and contralateral VCA. A similar result was shown by Kulesza (2014) and is consistent with the data on projections of the CR-ir neurons of the VCA in other species (Cant and Casseday 1986; Kil et al. 1995; Cant and Benson 2003).

### MNTB and Glycine in the SOC

In other species, the MNTB is the main source of glycinergic input to the SOC and we show clear evidence for glycinergic innervation of SOC in humans. What might be the source of that input? In other species there is evidence for glycinergic neurons in different periolivary nuclei, e.g. the lateral nucleus of the trapezoid body (Spirou and Berrebi 1997; Zacher and Felmy 2024), the LSO (Saint Marie et al. 1989), and the superior paraolivary nucleus (Zacher and Felmy 2024). Our data in humans are consistent with the idea that there are glycinergic periolivary neurons, but we have not directly identified these cells. In other species, glycinergic neurons have been identified with immunohistochemistry, however the available antibodies require glutaraldehyde in the fixative. The human material we have was fixed in formalin (see discussion in Baizer et al. 2024). It is possible, then, that in humans neurons with the functions of the MNTB are scattered around the MSO. This idea is also consistent both with observation of CR-ir afferent input to periolivary regions, allowing comparison of differences in the signals from the two ears, and with the evidence of glycinergic input to the MSO.

The results support the well-established idea that there are major species differences in the configuration and circuitry of the auditory brainstem. It is difficult to consistently identify an MNTB in Nissl sections. However, some of the scattered CB-ir neurons may subserve the functions of the MNTB. We add the SOC to the set of brainstem nuclei that show individual variability (Baizer et al. 2018b, 2021; Baizer 2014).

### Functional significance of individual differences

Individual differences in structure of the human SOC suggest that there might be correlated functional differences. The SOC are the first location in the auditory pathways in which information from the two ears converges on single neurons, an essential step in sound localization (Moore 1991; review in Grothe et al. 2010; Goldberg and Brown 1969). Is there evidence for individual differences in sound localization?

Many studies of human sound localization show variability among individual subjects (Lorenzi et al. 1999; Langendijk and Bronkhorst 2002; Recanzone et al. 1998; Kerber and Seeber 2012). Other studies have found differences between groups of individuals that differ in one or more parameters, for example, age (Dobreva et al. 2011; Freigang et al. 2015), sex (Savel 2009; Zündorf et al. 2011), sightedness (Gougoux et al. 2005; Lewald 2002), and handedness (Savel 2009).

Another critical way individuals may differ is in the efficacy of plasticity in sound localization. Plasticity is critical for recovery of localization ability in patients with impaired hearing who have received cochlear implants or hearing aids. Recovery can be facilitated by training (Du et al. 2015; Firszt et al. 2015; Luntz et al. 2002). There is evidence for individual differences in speed and degree of recovery of the ability to localize sound after implants (Kerber and Seeber 2012; Asp et al. 2011; Dunn et al. 2008; Beijen et al. 2007; Grieco-Calub and Litovsky 2010; Zheng et al. 2022).

A critical question is whether it is possible to correlate individual functional differences with structural differences. Anatomical studies require data on localization abilities of the human subjects whose brains are studied; unfortunately, such data are not available for the samples we have. Another approach uses imaging techniques to examine cortical regions that mediate localization (for example Maeder et al. 2001; Lewald et al. 2008); these techniques do not yet have the resolution to look at brainstem structures. Individual differences in motor abilities and skills are widely recognized and even celebrated (e.g. the Olympics). Differences in sensory abilities are less acknowledged and recognized. Exceptions include the gustatory skills of sommeliers (Bosker 2015) and the olfactory skills of perfume makers (Syme 2024). Differences in human motor and sensory skills may be associated with individual differences in the anatomy and physiology of the relevant neural pathways; understanding those differences remains a major challenge.

## Data Availability

All slides or images described in this paper can be made available to anyone by request to the corresponding author.
